# Do Women's Voices Provide Cues of the Likelihood of Ovulation? The Importance of Sampling Regime

**DOI:** 10.1371/journal.pone.0024490

**Published:** 2011-09-21

**Authors:** Julia Fischer, Stuart Semple, Gisela Fickenscher, Rebecca Jürgens, Eberhard Kruse, Michael Heistermann, Ofer Amir

**Affiliations:** 1 Cognitive Ethology Laboratory, German Primate Center, Göttingen, Germany; 2 Centre for Research in Evolutionary Anthropology, Roehampton University, London, United Kingdom; 3 Department of Phoniatrics and Pedaudiology, Georg-August Universität Göttingen, Göttingen, Germany; 4 Reproductive Biology Unit, German Primate Center, Göttingen, Germany; 5 Department of Communication Disorders, Sackler School of Medicine, Tel-Aviv University, Tel-Aviv, Israel; Roehampton University, United Kingdom

## Abstract

The human voice provides a rich source of information about individual attributes such as body size, developmental stability and emotional state. Moreover, there is evidence that female voice characteristics change across the menstrual cycle. A previous study reported that women speak with higher fundamental frequency (F0) in the high-fertility compared to the low-fertility phase. To gain further insights into the mechanisms underlying this variation in perceived attractiveness and the relationship between vocal quality and the timing of ovulation, we combined hormone measurements and acoustic analyses, to characterize voice changes on a day-to-day basis throughout the menstrual cycle. Voice characteristics were measured from free speech as well as sustained vowels. In addition, we asked men to rate vocal attractiveness from selected samples. The free speech samples revealed marginally significant variation in F0 with an increase prior to and a distinct drop during ovulation. Overall variation throughout the cycle, however, precluded unequivocal identification of the period with the highest conception risk. The analysis of vowel samples revealed a significant increase in degree of unvoiceness and noise-to-harmonic ratio during menstruation, possibly related to an increase in tissue water content. Neither estrogen nor progestogen levels predicted the observed changes in acoustic characteristics. The perceptual experiments revealed a preference by males for voice samples recorded during the pre-ovulatory period compared to other periods in the cycle. While overall we confirm earlier findings in that women speak with a higher and more variable fundamental frequency just prior to ovulation, the present study highlights the importance of taking the full range of variation into account before drawing conclusions about the value of these cues for the detection of ovulation.

## Introduction

An understanding of human sexuality is crucial for the evolutionary analysis of hominine morphology, life history, behavior and social organization [1]. A key issue in this respect is the information about women's fertility that is available to men. Unlike many nonhuman female primates, which exhibit obvious signals of their ovulatory phase such as brightly colored sex skin or large sexual swellings [2], ovulation in women has until recently generally been viewed as being concealed [3], [4]. Concealed ovulation and women's extended sexuality have been related to the development of the monogamous social system, which is prevalent in most human cultures [1]. However, recent evidence indicates that more subtle visual or olfactory cues might provide cues of the fertile phase of women, which are potentially salient to men. With respect to visual cues, the asymmetry of soft tissue traits such as ears and fingers is lowest around the time of ovulation [5], while female facial attractiveness significantly increases during the fertile phase of the cycle [6]. In addition, women appear to dress more attractively [7] and provocatively [8] during their ovulatory period. In terms of olfactory cues of fertility, a number of studies have found that men are more attracted to the scents of women in their likely fertile period than outside of this time [9]–[11]. For instance, female lap dancers were reported to receive significantly higher tips during the fertile phase of their cycle, compared with the luteal phase [12], suggesting that a combination of factors including smell, appearance and possibly movement patterns may change in relation to cycle phase [13].

Despite the growing evidence that visual and olfactory modalities may be important as indicators of ovulation in humans, results regarding the acoustic modality, that is whether women provide cues of reproductive state in their voices, are scarce. Numerous studies revealed that voices are a rich source of information, for instance with regard to gender and age [14], overall variation in size [15], developmental stability [16] or emotional state [17]. A recent study demonstrated that attractiveness ratings of women's voices varied across the menstrual cycle, with higher attractiveness ratings for recordings made during high-conception risk [18]. From a physiological point of view, this finding may not be so surprising. The vocal fold mucosa contains specific receptors for sex hormones [19]–[21]. During puberty, sex hormones such as estrogen, progesterone and testosterone affect the morphology of the larynx [22]. Furthermore, a series of studies by Amir and colleagues [23]–[26] has shown that women who take mono-phasic oral contraceptives, and therefore maintain a steady hormonal climate, exhibit improved vocal qualities and stability, in comparison with women who do not use oral contraceptives. Finally, women's voices undergo a significant change after menopause [22], [27], [28] and following hormone replacement therapy [29], [30]. Taken together, these findings suggest a link between hormone levels and histological changes in the vocal folds [29], which in turn may affect their mass, viscosity and tension, and therefore modify their oscillation properties [31], [32].

Bryant and Haselton [33] provided evidence for acoustic variation in relation to cycle stage. They obtained voice recordings from 69 subjects in high- and low-fertility periods as estimated by average cycle length. Ovulation was ascertained by detection of a urinary LH surge during the high-fertility period using a commercially available urine test. Subsequently, a number of acoustic variables were measured from sustained vowels as well as spoken sentences. While there were no significant differences in vowel characteristics, women produced spoken sentences with a slightly, but significantly higher fundamental frequency (211 compared to 206 Hz on average) during high- as compared with low-fertility. What remained unclear, however, was how this change compared to variation in fundamental frequency across the entire cycle and in relation to a more direct assessment of timing of ovulation based on the continuous measurement of female reproductive hormones. This information is essential to understand how precisely voice characteristics indicate the ovulatory period. Further, it is important to clarify the link between fluctuations in sex hormones and acoustic characteristics in more detail, to provide a comparative perspective for equivalent studies in nonhuman primates [34], [35].

Based on these previous studies assessing the hormonal influence on voice characteristics and the differential ratings of female voices during the menstrual cycle [18], we predicted that voice characteristics should systematically vary during the female menstrual cycle and potentially provide information about the fertile phase. Since higher pitched voices in women have been reported to be more attractive [36], [37], and in light of the findings by Bryant and Haselton [33], we furthermore predicted that the fundamental frequency should be higher in the time with high-conception risk (i.e. the peri-ovulatory period), than at other times of the menstrual cycle. We complemented the analysis of the link between fertility and acoustic characteristics with a rating study in which male listeners were asked to indicate their preference for voice samples that were presented in a pair-wise fashion. We predicted that subjects would indicate a preference for the voice samples recorded during the period of high-conception risk compared to the other phases in the cycle.

## Results

**Figure 1:**
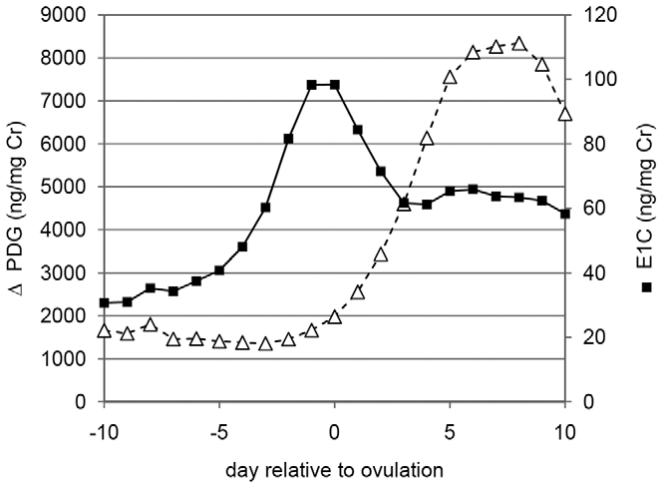
Variation in urinary sex hormone levels across the menstrual cycle. Average estrogen (E1G) and progestogen (PdG) values in the period of ten days preceding ovulation to ten days after the estimated day of ovulation. Values were compiled using data from 12 women for which we had complete hormone and acoustic data for the entire period, as well as from two women for which the first 2 days were missing.

The estrogen (E1G) and progestogen (PdG) profiles of the women in this study varied clearly and predictably across the cycle (Fig. 1). As expected for an ovulatory cycle, there was a notable estrogen peak just prior to ovulation, and a subsequent increase in progestogen values in the second half of the cycle in all subjects included in the analysis.

**Figure 2:**
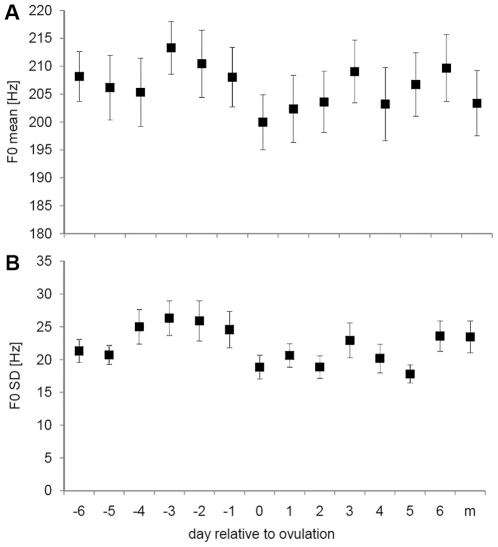
Variation in free speech voice characteristics. Mean (and s.e.m.) of (A) fundamental frequency and (B) standard deviation of the fundamental frequency of free speech segments, in relation to the day of ovulation and at the onset of menstruation (m).

In the free speech segments, the fundamental frequency varied marginally significantly between the different phases of the cycle (F_13,273_ = 1.56, P = 0.096, Partial Eta^2^ = 0.069, Observed Power = 0.842). The highest values of the fundamental frequency were observed in the days preceding ovulation, while the lowest values were observed during the period of ovulation (Fig. 2). Post-hoc comparisons (LSD) revealed significant differences between the days preceding ovulation and around ovulation (Table 1). There were also a number of non-significant differences. For instance, the days preceding ovulation did not differ from the days 3, 5 and 6 post-ovulation (Table 1). The standard deviation of F0 showed significant variation across the cycle (F_13,273_ = 2.14; P = 0.012, Partial Eta^2^ = 0.093, Observed Power = 0.952), and was significantly higher in the days prior to ovulation. There was no significant relationship between mean E1G and PdG levels and either of these two acoustic variables (GLM, P>0.2 for all variables).

**Table 1:** Results of post-hoc comparisons of variation in free speech on different days in the cycle.

day relative to ovulation	−6	−5	−4	−3	−2	−1	0	1	2	3	4	5	6	M
−6		0.641	0.518	0.225	0.598	0.980	0.054	0.170	0.290	0.837	0.251	0.742	0.720	0.256
−5	0.816		0.850	0.096	0.326	0.658	0.149	0.372	0.555	0.506	0.497	0.900	0.412	0.511
−4	0.166	0.110		0.070	0.251	0.534	0.223	0.497	0.696	0.402	0.633	0.759	0.322	0.653
−3	**0.050**	**0.030**	0.614		0.501	0.216	**0.002**	**0.010**	**0.025**	0.320	**0.020**	0.134	0.392	**0.019**
−2	0.078	0.048	0.739	0.865		0.581	**0.015**	**0.060**	0.119	0.750	0.099	0.404	0.863	0.100
−1	0.205	0.138	0.869	0.488	0.607		0.057	0.178	0.302	0.818	0.261	0.761	0.701	0.267
0	0.332	0.467	**0.021**	**0.004**	**0.007**	**0.026**		0.576	0.406	**0.035**	0.459	0.125	**0.023**	0.426
1	0.788	0.974	0.100	**0.026**	**0.043**	0.125	0.483		0.776	0.118	0.846	0.319	0.084	0.812
2	0.346	0.481	**0.024**	**0.005**	**0.008**	**0.030**	0.995	0.496		0.212	0.930	0.483	0.160	0.959
3	0.538	0.401	0.435	0.186	0.254	0.523	**0.116**	0.378	0.126		0.182	0.600	0.882	0.184
4	0.661	0.837	0.075	**0.019**	**0.031**	0.094	0.610	0.860	0.622	0.301		0.431	0.135	0.969
5	0.184	0.276	**0.009**	**0.001**	**0.003**	**0.011**	0.694	0.285	0.696	0.057	0.380		0.500	0.443
6	0.377	0.268	0.592	0.282	0.371	0.701	0.064	0.249	0.072	0.795	0.193	**0.030**		0.136
M	0.402	0.289	0.561	0.261	0.347	0.666	0.071	0.269	0.079	0.831	0.209	**0.033**	0.963	

Above diagonal: results for F0; below diagonal results for F0 SD. M: day 2 after onset of menstruation. Significant differences at the corrected level are indicated in bold.

**Figure 3:**
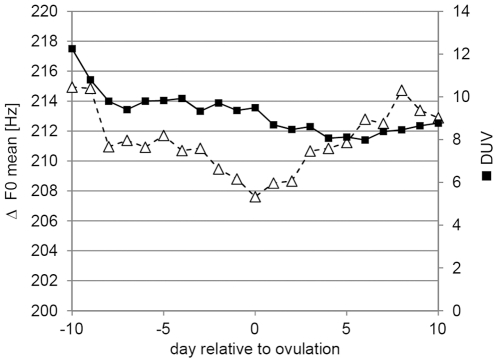
Variation of sustained vowel voice characteristics across the menstrual cycle. White triangles: mean fundamental frequency (F0); black squares: degree of unvoiceness (DUV); for the vowel ‘a’.

For the sustained vowels, the fundamental frequency was highest at the beginning of the cycle, dropped somewhat in the ovulatory period and then increased again (Fig. 3). This variation, however, was not significant (Table 2). The only significant variation between the phases was found for the noise-to-harmonic ratio (NHR) and degree of unvoiceness (DUV) (Fig. 3). Post-hoc tests revealed that DUV differed in the menstrual phase from all other phases, while NHR differed significantly only between menstruation and ovulation (LSD test). All other acoustic variables revealed no significant variation (Table 2). Neither estrogen nor progestogen values predicted changes in any of the acoustic variables (all P>0.2; data not shown). There was also no effect of recording order on any of the acoustic variables (all P>0.4).

**Table 2:** Acoustic characteristics in the different phases of the menstrual cycle with results of statistical tests.

		Cycle Phase							
Acoustic Variable	Follicular	Pre-Ovul	Ovulatory	Luteal	Menstr.	F	P	Partial Eta^2^	Observed Power
Fundamental frequency	213±3	212±3	211±3	214±3	214±4	0.90	0.464	0.011	0.285
F0 maximum	252±4	251±4	249±5	254±4	256±5	1.63	0.165	0.020	0.502
F0 minimum	185±3	185±3	185±3	187±3	185±3	0.35	0.840	0.005	0.131
F0 SD	5.3±0.3	5.4±0.3	5.0±0.2	5.5±0.3	5.6±0.4	1.32	0.262	0.017	0.412
Degree of unvoiceness	8.2±0.9	8.0±0.9	7.2±0.6	7.1±0.8	9.1±1.1	2.89	0.022	0.036	0.779
Noise-to-harmonic ratio	0.139±0.004	0.138±0.004	0.132±0.003	0.141±0.005	0.147±0.007	2.50	0.042	0.031	0.710
Shimmer	4.3±0.3	4.6±0.3	4.2±0.2	4.3±0.3	4.5±0.3	0.86	0.487	0.011	0.274
Jitter	2.1±0.1	2.1±0.2	1.9±0.1	2.0±0.1	2.1±0.2	1.85	0.119	0.023	0.559

Mean and s.e.m for N = 23 women and results of the statistical analysis the effect of cycle phase on different acoustic variables.

GLM with subject and vowel as random factor, and menstrual cycle phase as fixed factor; df = 4,313 for all analyses. Partial Eta^2^ provides an overestimate of the actual effect size; observed power gives the probability that the test statistic is greater than the critical value under the alternative hypothesis.

**Figure 4:**
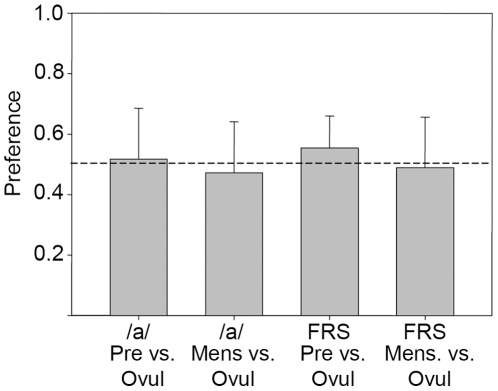
Degree of preference for female voices in different periods of the cycle. Samples recorded on the estimated day of ovulation (‘Ovul’) were compared with those recorded 3 days prior to this day (‘Pre’), and with those recorded on the second day of the menstrual cycle.

In the perceptual preference tests, British heterosexual males who did not speak German rated the free speech samples recorded three days prior to estimated ovulation to be, marginally significantly, more attractive than those recorded in the middle of the ovulatory period (t_27_ = 2.662, p = 0.013). There were no significant differences between attractiveness ratings during the period of ovulation and at the onset of menstruation (t_27_ = −0.377, p = 0.709; see Fig. 4). For the sustained vowels, we found no difference in preference (vowel /a/ pre-ovulation vs. ovulation t_27_ = 0.493, p = 0.626; ovulation vs. menstruation t_27_ = −0.891, p = 0.381).

## Discussion

Our analysis revealed higher than average F0 values in the free speech segments in the pre-ovulatory period, and a significant drop around the day of ovulation. Similarly, women produced speech with greater variability in F0 in the days preceding ovulation, compared to the time around ovulation. The analysis of the sustained vowels revealed no significant variation in fundamental frequency across the cycle. The only two parameters that exhibited significant variation were the degree of unvoiceness (DUV) and the noise-to-harmonic ratio (NHR), which revealed significantly higher values during menstruation.

The finding that F0 is higher prior to ovulation mirrors the results of a previous study, which reported an increase in fundamental frequency in free speech in the pre-ovulatory period [33]. This finding was taken as an indicator of the fertile phase of the women. Our analysis however suggests that the predictive value of the fundamental frequency is limited by the extended variation of F0 in the luteal phase of the cycle. Specifically, the days preceding ovulation did not differ from the post-ovulatory period. Moreover, the explained variance was extremely low. Even more puzzling is the finding that the fundamental frequency dropped significantly around ovulation: 17 of the 23 subjects produced a lower voice on the estimated day of ovulation compared to the previous day. On that estimated day of ovulation – including a margin of error of one day – however, the conception risk is as high as in the preceding two days [38], and thus, if there had been a selective pressure to broadcast information about fertility, this variation would be hard to explain. Overall, our study highlights the limitations of comparing only two phases in the menstrual cycle: while the observed pre-ovulatory increase in fundamental frequency is suggestive, the inclusion of the luteal and the menstrual phase questions whether this variable can indeed be used as an indicator of female fertility.

Despite a lack of clear cues to the pre-ovulatory period in the sustained vowels, we found significant changes in voice characteristics during the time of menstruation, particularly in noise-to-harmonic ratio (NHR) and degree of unvoiceness (DUV). Both variables are associated with vocal fold vibration patterns, which appear to be less stable during the time of menstruation. This leads to a less harmonic vocal pattern, which is reflected by the NHR parameter; and a higher degree of voice breaks, which is reflected by the DUV parameter. While these results indicate a relationship between endocrine milieu and voice characteristics, the underlying physiological mechanisms remain to be explored in more detail. It was previously shown that breast tissue, for example, increases in water content with rising progesterone levels, with water content peaking about 2–4 d after the progesterone peak [39]. Possibly, the increased hoarseness during menstruation (which is evident in the higher NHR and DUV values) is related to a similar increase in water content in vocal fold tissue.

Our perceptual experiments revealed a marginal preference for the free speech samples recorded in the period with the highest conception risk. One problem with using free speech is that content could be a major confound. This was at least partly alleviated by the fact that German speech was presented to British listeners who did not speak German. Free speech certainly varies more strongly in relation to changes in mood and motivation than sustained vowels, and perhaps also more than standardized sentences. The lack of preference for sustained vowels recorded in any of the three periods suggests that listeners indeed pay more attention to variation at the level of the entire sentence, i.e. prosody, than to changes that become evident at the level of the phoneme, such as the degree of unvoiceness and noise-to-harmonic ratio. The effect of the mode of speech production warrants further attention, as it was shown that reading scripted texts results in a higher F0 than spontaneous speech [40].

In previous experiments [33], pairs of selected speech samples where the high-fertility period revealed a higher pitch were presented to listeners. The average difference was 13 Hz. Although listeners accurately identified the higher-pitched sample as higher, at above chance levels, the hit rate was low with only 55% correct [33]. Note that in that study, subjects were not asked to judge attractiveness, but simply whether they perceived a change in pitch. Our results provide a more realistic assessment, as we did not pre-select the samples according to their pitch, and we directly asked which of the samples was considered to be more attractive. Altogether, the preference was relatively weak (Fig. 4).

Overall, the results regarding the relation between pitch and attractiveness indicate that the pattern may be more complicated than initially suggested. A recent study by Hughes and colleagues [41] provided evidence that women lower their voice pitch when talking to attractive men, and that such changes may increase the perceived pleasantness of the voice. Their study challenges the idea that pitch of the female voice is simplistically related to perceived attractiveness. Another study, however, found that women talked with a higher voice to men whom they considered to be attractive [42]. In both studies, the effect sizes were relatively low. Nevertheless, these findings may suggest that the observed variation in pitch is related to social factors and possibly a cultural phenomenon. Moreover, the fundamental frequency is only one component of voice attractiveness. To complicate matters further, a recent study revealed no preference for higher pitched female voices. Attractiveness ratings were relatively stable for an F0 between 220 and 260 Hz, and only decreased for voices with very low or very high F0 [43].

Our study contributes to the growing body of evidence that a number of cues may vary during the female cycle [5], [6], [9]–[12], but that the variation may not be sufficiently specific to allow for identification of the fertile phase. A study in Barbary macaques measured day-to-day variation in estrogen and progestogen values and assessed whether they predicted the acoustic structure of female Barbary macaque copulation calls [35]. The observed variation in acoustic variables across the cycle did not reflect short-term changes such as the peak in estrogen occurring around the timing of ovulation in the macaques, suggesting that the observed principle of gradual changes in acoustic structure may apply across species. In addition, subtle changes in fertility-related signals may only be salient to a partner who is familiar with the signaler, as was observed in rhesus macaques [44].

Taken together, our study corroborates earlier findings regarding the increase of the fundamental frequency just prior to ovulation [33], and the preference for a higher pitch in women's voices [18]. At the same time, a note of caution is warranted, as the overall variation across the cycle indicates that pitch changes may be a poor predictor of actual fertility. Future studies assessing cues to reproductive state should therefore include samples throughout the female cycle. Moreover, we feel that the results of laboratory tests of human attractiveness - such as the observed preference of just about 55% for female faces in the fertile compared to the luteal phase [6] - need to be considered carefully when looking at real world contexts: while the results may be statistically significant, the biological significance [45] in actual mate choice is far from clear.

## Materials and Methods

### Ethics Statement

The study was approved by the ethics committee of the Medical faculty of the Georg-August-University Göttingen (application number 16/2/05). Subjects provided written informed consent regarding their participation in this study.

### Subjects

We recruited young female candidates for participation in the study through advertisement at university switchboards and word-of-mouth propaganda in a middle-sized German university town. Potential candidates were asked to fill in an anamnesis questionnaire, in which the following information was collected: typical menstrual cycle length [days], birth year, height [cm] and weight [kg], history of voice or speech training or singing lessons, vocal activity (e.g. working in a call centre), overall physical activity and health status, smoking habits and drug use. The following criteria led to exclusion from the study: reported history of speech or voice disorders; prior history of neurological disease; recent infections of the respiratory tract, asthma or regular medication; use of hormonal contraceptives during the last three months before study onset; medication containing steroid compounds; irregular menstrual cycles; smoking or working in an environment with heavy smoking; drug abuse; extreme BMI. The remaining candidates were examined by a laryngologist using a KS 4200/S laryngo-stroboscope (Labor Dr. Timcke Hamburg, Germany), and an rpSzene video documentation system (Rehder und Partner, Hamburg, Germany). All participants had normal larynges, with both vocal folds presenting normal and symmetric vibratory pattern with no pathological findings and with complete glottal closure during phonation. All voices had a normal hoarseness diagram (Göttingen hoarseness diagram using the software lingWAVES, Forchheim, Germany) [46], [47], and were perceptually judged as normal by a speech therapist.

Of the 33 women who eventually entered the study, 10 had to be excluded because they had anovulatory cycles during the sampling period (N = 7), became pregnant (N = 1), dropped out due to personal reasons (N = 1) or did not collect the required daily urine samples during the peri-ovulatory period, such that timing of ovulation in them could not be assessed reliably (N = 1). The final study set, thus, comprised 23 women with a mean age of 22 years (range 18–26 years), a mean BMI of 21.0 (18.0–25.0) and a reportedly regular menstrual cycle length of 29.1 d (24–32 d). After completion of the data collection, participants were paid € 100 (plus a € 50 bonus, see below).

### Data sampling

**Figure 5:**
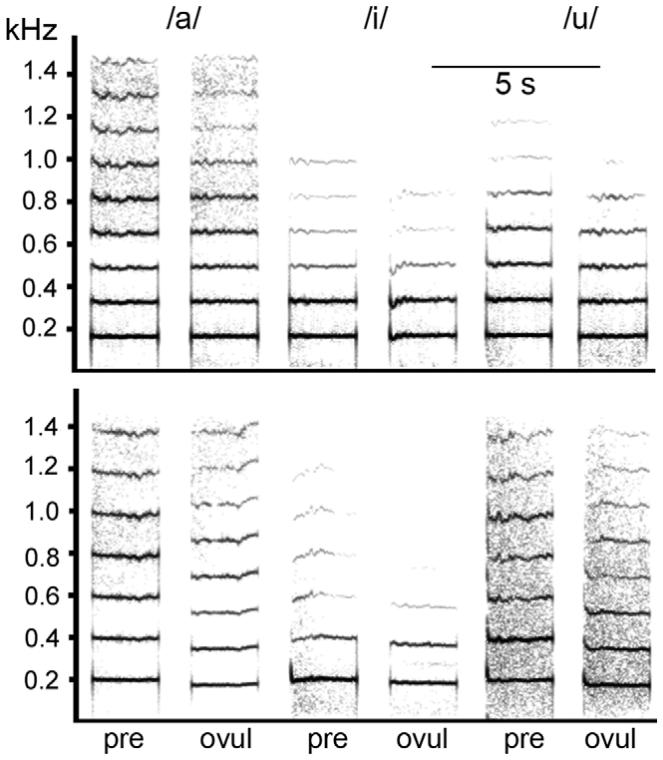
Variation in sustained vowels in relation to cycle phase. Spectrographic displays of the vowels /a/, /u/, and /i/ for two women in the preovulatory (pre-) and peri-ovulatory (ovul) period. Upper panel: this subject revealed no major variation in the two phases. Lower panel: this subject revealed a drop in F0 in the ovulatory period of about 20 Hz. Frequency on the y-axis and time on the x-axis. Sampling frequency 3200 Hz, FFT settings: length: 512 pt, frame 100%, Hamming window, frequency resolution 6 Hz, temporal resolution 40 ms (75% overlap).

Ten women began sampling on the first day of menstruation; six began about a third into the cycle (mean: 9.5 days after last onset of menstruation) and seven at the beginning of the last third of the cycle (19.4 days after last onset of menstruation). Data collection took place in subjects' homes. Subjects were requested to collect urine first thing in the morning (see below). Next, they were instructed to drink one glass of water and then read aloud ten lines of text given to them, to control the amount of vocal activity before voice recording began. They then started the voice recordings using a Marantz PMD 660 Flashcard-Recorder or a Mayah Flashman Harddisk-Recorder, with a sampling rate for recording of 48 kHz (16 bits). Sennheiser pc150 headset microphones were used to ensure a fixed distance of approximately 5 cm between the microphone and the corner of the mouth. Before the beginning of the sampling period, the women were instructed how to use the recording equipment and in particular how to adjust the recording level to avoid saturation and clipping of the sound file. Women were instructed to perform the recordings alone, and no other voices or sounds were to be heard on any of the recordings. As an incentive, we informed them that a € 50 cash bonus would be paid if the recording quality met the requirements (all did). After the first three days of sampling, the research assistant checked the quality of the recordings. The women recorded the following speech samples: (1) one min of free speech (comments on weather, plans for the day etc.; of these, ten 4 s segments were extracted and the mean F0 and the SD across the segment were analyzed); (2) sustained production of the vowels /a/ (as in ‘bar’), /i/ as in ‘bee’, and /u/ (as in ‘you’) (Fig. 5), each six times, read from a table in which vowels were presented in a randomized order differently for each day of the data collection period. Women were asked to sustain vowel production for about 5 s. Each single recording session lasted approximately five minutes.

### Acoustic analysis

Prior to performing the acoustic analysis, all recordings were normalized for amplitude. Then, F0 contours within each recorded sample were examined manually, by displaying the sound signal on the computer monitor, with simultaneous auditory and visual evaluation of the signal, to ensure that no octave errors occurred during the preliminary stage of the analysis.

Firstly, we determined variation in F0 and STD of F0 in a 4 s segment of free speech for all 23 subjects for the week preceding and following the estimated day of ovulation (N = 13 days) plus one day after the onset of menstruation. The mean F0 and the SD of these segments were measured using Avisoft-SASLab Pro Version 4.52 (Avisoft Bioacoustics, Berlin, Germany). For this purpose, spectrograms were created (sampling frequency of 2.2 kHz, Hamming window and 98.43% overlap) with a 1.1 kHz frequency range, a time resolution of 7 ms, and a frequency resolution of 2 Hz, from which the F0 was measured by hand every 0.2 s using the Free reticule cursor.

Secondly, from the sustained vowels, the following acoustic parameters were measured using the software package Multidimensional Voice Profile MDVP (Kay Elemetrics Corporation, Lincoln Park, NJ): mean fundamental frequency (F0) [Hz], maximum and minimum F0 [Hz], standard deviation of F0 [Hz], degree of unvoiceness (DUV), i.e. the ratio of the unvoiced part of the signal to the whole signal; noise-to-harmonic ratio (NHR), i.e. the ratio of non-harmonic components of the voice signal (noise) to the harmonic components; Jitter (cycle-to-cycle frequency perturbation), and Shimmer (cycle-to-cycle amplitude perturbation).

The values obtained from the individual recorded segments (i.e., spoken sentences or isolated vowels) were averaged by type of signal, within each recording day. This was performed to obtain a reliable representation of the voice signal. For example, the acoustic measurements obtained from the six repeated productions of the vowel /a/ within each recording day were averaged, for each participant. This yielded a mean value for each acoustic parameter, which was then submitted to the statistical analyses.

### Hormone analysis

In order to determine the day of ovulation, the women collected daily first-morning urine specimens (from the mid-stream) for one full cycle. A 2 ml aliquot of the urine sample was transferred into a (bisphenol A-free) Eppendorf cup and stored frozen until hormone analysis. The research assistant collected the samples regularly and transferred them to the laboratory for analysis. The day of ovulation was determined based on the distinct changes in estrogen and progesterone secretion during the peri-ovulatory phase. These changes are reflected in the concentrations of the urinary metabolites of these hormones, namely estrone-3-glucuronide (E1G) and pregnanediol-3-glucuronide (PdG), respectively [48], [49]. Based on the measurement of these two steroids and according to [49], we identified the day of ovulation by applying, in each cycle, an algorithm which utilizes the ratio of the two hormones to determine the “day of luteal transition” (DLT). This day marks the shift from production of follicular estrogen to luteal progesterone during the peri-ovulatory phase of the women's menstrual cycle and indicates the time when the estrogen/progesterone ratio begins to drop following ovulation [49]. The algorithm has been validated against the surge of urinary LH by showing that DLT is not only highly concordant with the day of urinary LH [49], [50], but that it is also as accurate as the latter in estimating the time of ovulation in women [51], [52]. Therefore, and in accordance with previous studies that have used this approach successfully to estimate ovulation date [53], [54], we defined the day of luteal transition in our study subjects as representing the most likely day of ovulation. Due to the variability between DLT and the urinary LH peak [49], this estimated day of ovulation may include an error of ±1 day. This algorithm approach is considered to be the most appropriate and accurate method for timing of ovulation in women [49], [52], particularly since a urinary LH peak required alternatively for timing ovulation is not detectable in up to 44% of cycles [49], [55]. Moreover, daily ultrasound monitoring of follicular rupture as the gold standard is not feasible under non-clinical conditions.

Concentrations of urinary E1G and PdG were measured directly in unextracted samples using enzymeimmunoassays as described by Heistermann and Hodges [56]. For hormone analysis, urine samples were diluted 1∶200 (E1G) and 1∶800 (PdG) in 0.04 M phosphate buffered saline and 50 µl aliquots were taken in duplicate to assay. Intra- and inter-assay coefficients of variation of high and low value quality controls were 4.2% (n = 16) and 4.6% (n = 22) (high) and 4.9% (n = 16) and 5.3% (n = 22) (low), respectively for E1G. Corresponding figures for PdG were 5.2% (n = 16) and 5.9% (n = 22) (high) and 8.5% (n = 16) and 11.9% (n = 22) (low), respectively.

### Statistical analysis of speech samples

The free speech data were analyzed using a Linear Mixed Model, with subject as random and cycle day as fixed factor, applying the restricted maximum likelihood estimation (REML). For 8 subjects, only 13 of the 14 repeated measures were available and for one subject only 12 data points. For the remaining 14, all 14 data points were available.

For the sustained vowels, we used two approaches. Firstly, we defined specific periods in the cycle and compared the average value for those periods. The period of days 7 to 5 before ovulation was defined as the follicular phase, the following three days (4 to 2 before ovulation) as pre-ovulatory, the three days around ovulation as ovulatory, the days 5–7 post ovulation as post-ovulatory (luteal phase), and the days 1–3 after onset of menstruation as menstruation (N = 5 phases). We then calculated the mean values for each variable and subject for each of the three vowels and phase (N = 23 subjects). We used Linear Mixed Models to assess the influence of the cycle stage on acoustic features, with phase as fixed factor and subject ID and vowel category as random factors, applying the restricted maximum likelihood estimation (REML). Secondly, we examined the day-to-day acoustic variation from the period of 10 days before to 10 days after ovulation. This allowed us to explore the link between hormone levels and putative acoustic variation in more detail. Because of the sampling scheme, we were not able to collect continuous data for all of the women in the study so that this analysis is restricted to 14 subjects. To minimize the effect of random fluctuations, we applied a running mean procedure (window size = 3), including days −11 and 11, so that we obtained smoothed values for both the predictor and the dependent variables for the days −10 to 10. We then used the Generalized Estimating Equations procedure, with subject as random factor, PdG, E1G and recording day as co-variates and the respective acoustic variables as dependent variables. All statistical analyses were conducted with PASW 18 (SPSS Inc.). We refrained from post hoc power analyses of non-significant results because these do not provide more information than the p value itself [57]. Instead, we provide assessments of the effect size and observed power.

### Preference tests

Voice samples used in these tests were recorded from ten different women at one of three time periods: ovulation (i.e. on the estimated day of ovulation), pre-ovulation (3 days before the estimated day of ovulation) or the beginning of the cycle (the second day of the menstrual cycle). Because the estimated day of ovulation has a margin of error of one day, the selection of the samples for the first two of these periods should be considered as representative of the ‘ovulatory period’ and the ‘pre-ovulatory period’, respectively. Two different sample types were used: the vowel /a/ and a 5 s section of free speech. For each female subject, four exemplars of each sample type for each time period were used in order to reduce issues of pseudoreplication. During a testing session, there were four choice tests, comparing (1) vowel /a/, ovulation vs. pre-ovulation, (2) vowel /a/, ovulation vs. beginning of the menstrual cycle (3) 5 s of free speech, ovulation vs. pre-ovulation, (4) 5 s of free speech, ovulation vs. beginning of the menstrual cycle.

Twenty eight heterosexual male English subjects were recruited for the study from the student population at Roehampton University. They were informed prior to testing that they would be taking part in a study of vocal attractiveness, but no further information on the nature of the study was provided. The subjects did not speak or understand German. During testing, subjects sat alone in a small, windowless room and listened to voices played from a Dell Optiplex desktop computer through Sennheiser HD280 PRO closed headphones. Within each of the four choice tests, there were ten trials (one for each female from whom voice recordings were used in the study). In each trial, the subject heard two voices, presented one after the other with a gap of 5 seconds in-between; the voices in each trial pair were from the same woman. The order of presentation of the two voices for each woman was decided at random, and the order in which voices of the ten women were presented within tests was also randomized. For each trial, subjects were asked to mark on a paper check sheet which of the two voices in the pair they found more attractive, i.e. this was a forced choice design.

For each subject in each of the four choice tests, the proportion of trials in which they scored the ‘ovulation voice’ as more attractive was then calculated. For each choice test, the mean of this proportion was compared to the chance value of 0.5 in a one sample t-test. As there were four choice tests carried out, a Bonferroni correction was made, such that the critical level of significance was set at 0.0125.
